# Smart Molecular Recognition: From Key-to-Lock Principle to Memory-Based Selectivity

**DOI:** 10.3389/fchem.2019.00933

**Published:** 2020-01-21

**Authors:** Askar K. Gatiatulin, Marat A. Ziganshin, Valery V. Gorbatchuk

**Affiliations:** Department of Physical Chemistry, A. M. Butlerov Institute of Chemistry, Kazan Federal University, Kazan, Russia

**Keywords:** molecular recognition, selectivity, inclusion compound, clathrate, phase transition

## Abstract

The formation and decomposition of inclusion compounds with a solid-solid phase transition may be very selective to the guest molecular structure. This selectivity may function in essentially different ways than defined by the classical concept of molecular recognition, which implies the preferential binding of complementary molecules. Solid inclusion compounds may take part as an initial or/and final state in several processes of different types summarized in this review, which selectivity is boosted by cooperativity of participating molecular crystals. Some of these processes resemble switching electronic devices and can be called smart giving practically absolute molecular recognition.

## Introduction

Molecular recognition of neutral molecules is one of the key problems in chemical technologies and in analytical and biotechnological applications (Reinhoudt, 2013; Persch et al., 2015; Shu et al., 2018). To reach a sufficient selectivity, host compounds with very complex structure are synthesized (Ariga et al., 2012; Zhang et al., 2019) to fit the well-known key-to-lock concept of molecular recognition formulated by Fischer (1894). This concept later developed in supramolecular chemistry is based on complementarity of two interacting molecules, where the host interacts with guest cooperatively through several more or less strong coordinate, donor-acceptor, and hydrogen bonds having a specific spatial arrangement (Joyce et al., 2010; Sonnenberg et al., 2012). The most studies of molecular recognition are conducted in liquid solutions (Ariga et al., 2012; Persch et al., 2015; Shu et al., 2018; Zhang et al., 2019) and perform a sufficient selectivity only if guest forms at least two such bonds with host (Yao et al., 2018).

This review describes the possible alternatives to the classical key-to-lock principle with a higher selectivity of molecular recognition. These alternatives are based on cooperativity of phase transitions, which adds up the small differences in molecular structure of different included guests. Some of the described recognition principles can be called smart because they resemble the function of electronic devices.

**Figure 1:**
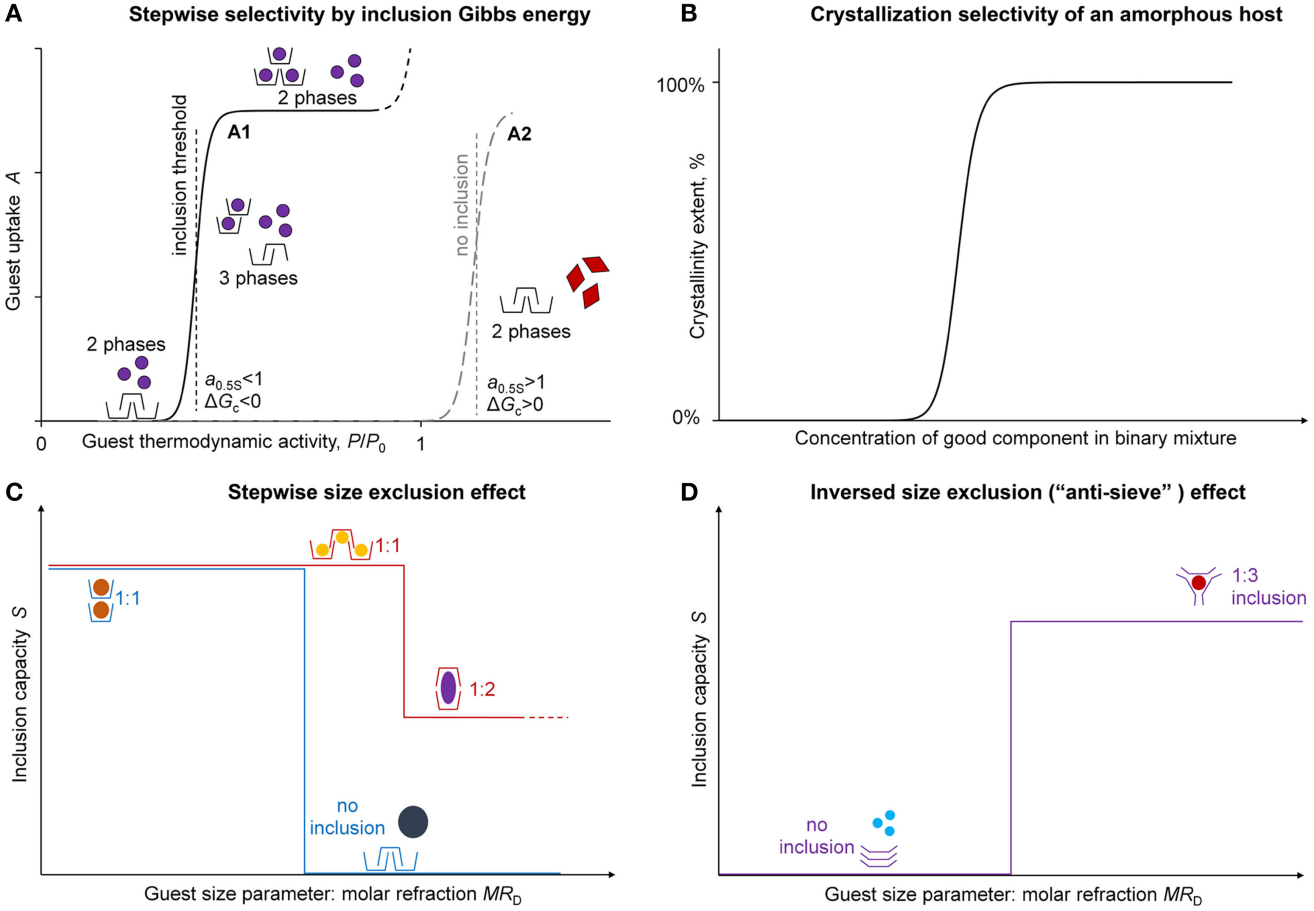
Stepwise inclusion selectivity of solid hosts. **(A)** Stepwise selectivity by inclusion Gibbs energy; **(B)** crystallization selectivity of an amorphous host; **(C)** stepwise size exclusion effect; **(D)** inversed size exclusion effect (“anti-sieve”).

Quantitatively, the cooperativity of phase transition at guest inclusion by solid host can be seen in a stepwise sigmoidal shape of guest sorption isotherm (Gorbatchuk et al., 1997a; Dewa et al., 1998). According to the Gibbs phase rule, a sorption isotherm in system with two independent components (guest and host) should have a threshold concentration, vapor pressure or thermodynamic activity of guest corresponding to formation of three phases of guest, host,and clathrate (inclusion compound) at constant temperature, Figure 1A (Gorbatchuk et al., 2002). Below this threshold activity, the guest is not included, and below and above this threshold the composition of the solid phase does not change.

In solid state, this phase transition is observed if the initial host is non-porous (Gorbatchuk et al., 2002). If the host has a permanent porosity combined with flexible structure, like that of some metal organic frameworks (MOFs) (Hiraide et al., 2016; Engel et al., 2017) or silicalites (DeJaco et al., 2019), the initial part of sorption isotherm may have the shape of Langmuir isotherm followed by a sigmoidal step. This step is called the gate-opening or breathing (Afonso et al., 2012; Lee et al., 2019). A similar cooperative phenomena were observed for biological objects, e.g., for oxygen binding by aqueous solution of hemoglobin (Yuan et al., 2015).

The sigmoidal isotherms of guest inclusion by solid host and related cooperativity of guest release from the inclusion compound may boost the selectivity of these processes. Depending on the initial and final states of host, several specific types of selectivity may be observed, which are described in this review.

## Crystallization Selectivity of Amorphous Host

Selectivity of guest inclusion may be visualized if the initial state of host is amorphous. The amorphous state is a high-energy state, so its transition to the crystalline state may be spontaneous (Faizullin et al., 2019). The activation of this process with guest vapors may be selective. Such selectivity was observed visually for a compact glass of calixarene (Gataullina et al., 2015, 2017) and using an atomic force microscopy for thin amorphous films of dipeptides (Ziganshin et al., 2015). Amorphous dipeptides may have three options in contact with guest vapors depending on the guest molecular structure: (1) crystallization, (2) gel formation, (3) intact host morphology (Ziganshin et al., 2017).

The amorphous calixarenes in the form of a compact transparent glass can be used to detect visually the composition of a binary guest mixture, where only one (good) component has an ability to induce the host crystallization. The mixture should have the concentration of this guest above a certain threshold value for this crystallization to be apparent, Figure 1B. For example, glassy *tert*-butylthiacalix[4]arene derivative crystallizes in contact with vapors of the aqueous solution of ethanol if its concentration is above 24 vol.% (Gataullina et al., 2015). The glass of the same calixarene in another conformation allows detecting 1% vol. of benzene in hexane (Gataullina et al., 2017). A similar crystallization behavior was observed for glassy polymers (Gao et al., 2012), which have a less pronounced concentration threshold for the good component in binary solvent due to the incomplete crystallization.

## Selectivity by Capacity and Gibbs Energy of Guest Inclusion

The guest inclusion by the host with the phase transition complicates much the structure-property relationships for this process. The related selectivity can be described using approximation parameters of sigmoidal isotherms of guest inclusion, Figure 1A. These isotherms may be fitted with Hill equation adjusted to “guest uptake *A* vs. relative vapor pressure *P/P*_0_” coordinates (Gorbatchuk et al., 1997a):

(1) $$\begin{array}{r} A=SC{\left(P/P_{0}\right)}^{N}/\left[1+C{\left(P/P_{0}\right)}^{N}\right] \end{array}$$

where *S* is guest contents in a saturated inclusion compound (clathrate) in mol of guest per 1 mol of host, *C* is a sorption constant, *N* is a cooperativity parameter, which in ideal case of phase transition should have an infinitely high value, *N* → ∞. The integration of sigmoidal sorption isotherms fitted by this equation gives the inclusion Gibbs energy Δ*G*_c_ of guest transfer from its pure liquid or solid state to the saturated inclusion compound (Gorbatchuk et al., 1999a):

(2) $$\begin{array}{r} \Delta G_{c}=RT\int_{0}^{1}\text{ln }\left({P/P}_{0}\right)\text{d}Y \end{array}$$

(3) $$\begin{array}{r} \Delta G_{c}=RT\text{ ln}a_{0.5\text{S}}=-RT\;\left(\text{ln}C\right)/N \end{array}$$

Here *Y* = *A/S* is the extent of host saturation with guest, *a*_0.5S_ is the guest activity *P/P*_0_ at *Y* = 0.50.

The thermodynamics defined by Equations (1–3) means the stepwise selectivity of guest inclusion. If two guests have very small difference in molecular structure, but the first guest has sorption constant *C* slightly below unity and for the second one this parameter should be slightly above this level, only the first guest will be included, Figure 1A. As a result, a high selectivity of guest inclusion may be observed discriminating the close homologs. For example, *tert*-butylthiacalix[4]arene includes methanol from the vapor phase, but not ethanol (Galyaltdinov et al., 2012).

The same inclusion thermodynamics may produce a stepwise change in the guest inclusion capacity *S* at the variation of the guest molecular structure, Figure 1C. A good example is *tert*-butylcalix[4]arene including a lot of guests inside its molecular cavity (Ripmeester et al., 2006) with a regular stepwise size exclusion effect between the inclusion capacity *S* and guest molar refraction *MR*_D_, which is a good molecular size parameter (Gorbatchuk et al., 1999b). The exclusions are the guests, which can break the host intramolecular cyclic H-bond, like 1-butylamine (Udachin et al., 2002).

In those cases, where also interstitial guest inclusion is possible, the structure-property relationship for the host inclusion capacity *S* may be more complex. *tert*-Butylcalix[5]arene with such structure of inclusion compounds has a very irregular relationship between S and *MR*_D_ values (Ziganshin et al., 2007). The same was observed for diol host (Gorbatchuk et al., 2000), adamantylcalix[4]arene (Yakimova et al., 2008), and *tert*-butylcalix[6]arene (Safina et al., 2013).

Rather regular size exclusion effect may be expected for hosts with strong intermolecular H-bonding in their crystals. This was observed for dry hydrophilic receptors α-cyclodextrin (Gatiatulin et al., 2018) and β-cyclodextrin (Gatiatulin et al., 2016). In both cases, hydrophilic guests are included better than hydrophobic ones. In this relation, the inclusion selectivity of dry cyclodextrins is similar to those of dry glassy hydrophilic receptors like human serum albumin (Gorbatchuk et al., 1997b, 1999c), β-lactoglobulin (Mironov et al., 2003), and cross-linked polyacrylamide derivative (Gorbatchuk et al., 2004).

The second type of host selectivity to the guest size is an inverted size exclusion or “anti-sieve” effect, where the host prefers larger molecules, while the smaller are not included, Figure 1D. Such selectivity was observed for thiacalix[4]arene, which may include guests into the interstitial space formed by too many calixarene macrocycles where a sufficient driving force apparently needed to push them aside (Galyaltdinov et al., 2014).

The solid-phase transition at guest inclusion by solid host implies also the host selectivity by inclusion threshold of guest thermodynamic activity, and accordingly, by inclusion Gibbs energy Δ*G*_c_, Figure 1A. The range of the observed Δ*G*_c_ values depends much on the size of host cavity that does not require work to be created (Gorbatchuk et al., 2002; Gatiatulin et al., 2018). For example, for the *tert*-butylcalix[4]arene, which includes the most guests studied inside its molecular cavity (Ripmeester et al., 2006; Ramon et al., 2011), there is a significant variation in Δ*G*_c_ from −1.2 to −8.9 kJ/mol for different guests (Gorbatchuk et al., 2002). *tert*-Butylthiacalix[4]arene with the same type of guest inclusion but with a smaller effective cavity has the Δ*G*_c_ values from −0.4 to −2.0 kJ/mol (Gorbatchuk et al., 2002). *tert*-Butylcalix[5]arene (Ziganshin et al., 2007), adamantylcalix[4]arene (Yakimova et al., 2008), and β-cyclodextrin (Gorbatchuk et al., 2013), which may include guests into interstitial space of their crystal packing, have an intermediate position by this parameter: with Δ*G*_c_ less negative than −4.6, −3.6 and −3.8 kJ/mol, respectively. If the interstitial inclusion is possible, the higher values of inclusion capacity *S* corresponds mostly to the less negative Δ*G*_c_ values (Ziganshin et al., 2007; Yakimova et al., 2008).

This type of selectivity explains the described above stepwise size exclusion effect in the guest inclusion by solid hosts. When the guest molecule is too big for the host molecular cavity, the structure-property relationship may have two options. Either there is a stepwise change to no inclusion, e.g., for *tert*-butylthiacalix[4]arene (Gorbatchuk et al., 2002), or a stepwise change to a different packing pattern with a lower guest content observed for *tert*-butylcalix[4]arene (Gorbatchuk et al., 1999b). One should not compare the selectivity by inclusion Gibbs energy Δ*G*_c_ and the selectivity by host-guest association constants *K*_a_ in liquid solutions from NMR titration experiments, which may give a huge overestimation of *K*_a_ values (Gorbatchuk et al., 2017).

## Selectivity of Inclusion Irreversibility

**Figure 2:**
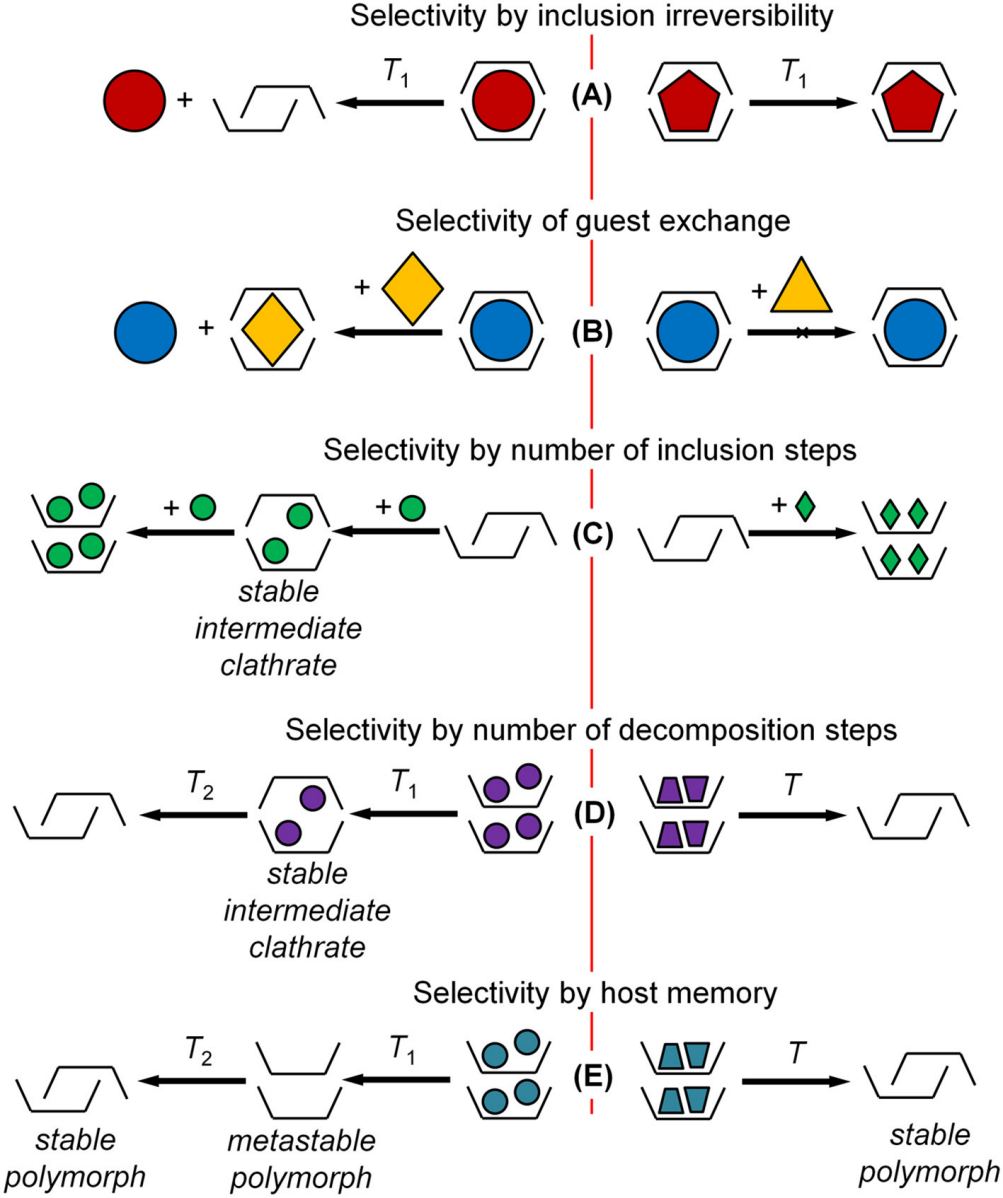
Specific types of molecular recognition using solid-solid phase transitions. **(A)** Selectivity by inclusion irreversibility; **(B)** selectivity of guest exchange; **(C)** selectivity by number of inclusion steps; **(D)** selectivity by number of decomposition steps; **(E)** selectivity by host memory.

Cooperativity of the guest inclusion process creates the additional selectivity options that can be used to enhance the efficient molecular recognition. Molecular structure of host and guest may have a strong impact also on the process of guest release, Figure 2A, e.g., in host regeneration of the sensor experiment. Being kinetically controlled through a strong sorption/desorption hysteresis (Dewa et al., 1998), guest release from the host-guest clathrate may have a different structure-property relationship than guest inclusion, which is under a thermodynamic control described above. This irreversibility may be detrimental in sensor experiments (Yakimova et al., 2008; Gorbatchuk et al., 2017), and the undesired history effect may be removed by high-temperature treatment of the host layer giving a normal sigmoidal shape of sorption isotherm by sensor unit (Matsuura et al., 2000).

The dependence of inclusion irreversibility on the guest molecular structure may be used to increase the selectivity of sensor experiment. A good example is the vapor sensor with a thin layer of adamantylcalix[4]arene on the quartz microbalance (Yakimova et al., 2008). The first run of this sensor experiment at 25°C and the second run after the host intermediate regeneration at 45°C by air purge give the sensor responses *R*_1_ and *R*_2_, respectively. The ratio of these responses *R*_2_/*R*_1_ is mostly different for different guests being a parameter of guest inclusion reversibility with *R*_2_/*R*_1_ ≤ 1. Using this parameter helps to increase the selectivity of single sensor analysis and to ensure recognition of more individual guests.

## Selectivity of Guest Exchange in Inclusion Compound

Along with the inclusion selectivity in binary host-guest systems, the selectivity of guest exchange in the solid phase of inclusion compound may be used for molecular recognition, Figure 2B. An efficiency of this exchange may depend on guest molecular structure in a different way than that of guest inclusion in binary system (Galyaltdinov et al., 2012; Amombo Noa et al., 2016). This gives an additional dimension to molecular recognition of guest compounds using the same host. For example, for thiacalix[4]arene (Galyaltdinov et al., 2014) and *tert*-butylthiacalix[4]arene (Galyaltdinov et al., 2012; Morohashi et al., 2019), the guest exchange increases the range of included compounds thus decreasing the inclusion selectivity. Still, this selectivity remains essentially stepwise. In some cases, the guest capable of inclusion in binary system cannot replace another guest in inclusion compound.

The guest inclusion by the host with a partial exchange of the already included water is a standard experimental procedure for solid hydrophilic hosts, such as native cyclodextrins (Ho et al., 2011, 2016; Gatiatulin et al., 2019) that do not include large hydrophobic guests in binary host-guest systems in the absence of water (Gorbatchuk et al., 2013; Gatiatulin et al., 2018). To activate this inclusion without water, the guest exchange in anhydrous inclusion compounds of cyclodextrins may be used (Gorbatchuk et al., 2013; Gatiatulin et al., 2014), which selectivity and efficiency depends much on molecular structure of the leaving guest. For example, 1-propanol and propionitrile cannot replace water in the saturated β-cyclodextrin hydrate but can exchange benzene, ethanol and acetonitrile in anhydrous clathrates with this host (Gorbatchuk et al., 2013; Gatiatulin et al., 2016).

## Selectivity by a Number of Steps of Guest Inclusion and Release

The geometric constraints for guest inclusion changing with the variation of guest content in inclusion compound (clathrate) may give another type of selectivity. This is the selective formation of stable intermediate clathrates, Figures 2C,D, which can be seen in two-step sorption isotherms (Ziganshin et al., 2007; Safina et al., 2010) and thermogravimetric (TG) curves (Yakimov et al., 2008). Sorption isotherms and TG curves of this type are relatively rare. So for *tert*-butylcalix[4]arene (Ziganshin et al., 2007), *tert*-butylcalix[5]arene (Ziganshin et al., 2007), and adamantylcalix[4]arene (Yakimova et al., 2008), two-step sorption isotherms or TG curves are observed for 2 out of 15, 3 out of 8, 2 out of 7 studied guests, respectively.

An example of absolute molecular recognition of benzene by a number of guest inclusion steps was observed for tetra(ethoxycarbonyl)methoxy thiacalix[4]arene (Safina et al., 2010). This calixarene performs a two-step inclusion only for benzene in experiments with quartz-crystal microbalance sensors, while all other studied guests are included in one step. This type of selectivity was observed also for benzene in mixtures with its close homologs. It fundamentally differs from the classical key-to-lock model.

## Smart Molecular Recognition: Selectivity by Host Memory for Previously Included and Released Guest

The irreversibility of guest inclusion and release with solid-solid phase transition can be a source of one more type of selectivity. This is selectivity of guest-induced polymorphism, which is a well-studied phenomenon used for screening of polymorphs (Braga et al., 2010; Petkune et al., 2012; Newman, 2013; Lee, 2014). A corresponding screening technique involves preparing the inclusion compound and removing the included guest (Lee et al., 2013; Gataullina et al., 2017). This is a smart process, where the host may remember molecular structure of a released guest by formation of a specific metastable polymorph (Gataullina et al., 2015).

An ideal case for molecular recognition is the host ability to form two polymorphs: stable and metastable ones, Figure 2E, where the metastable polymorph is formed after inclusion and release of only one guest and not of any other. Such an absolute selectivity for chloroform and methanol was found for *N*-(2-hydroxyethyl)carbamoylmethoxy) *tert*-butylthiacalix[4]arene (Safina et al., 2011) and for *tert*-butylthiacalix[4]arene (Galyaltdinov et al., 2012), respectively. For *tert*-butylthiacalix[4]arene, metastable polymorph is formed from its clathrate prepared only by solid-phase exchange of included 1,2-dichloroethane with methanol. In both cases, the formation of metastable polymorph can be detected by exothermic solid-solid phase transition of guest-free host in simultaneous experiment of TG and differential scanning calorimetry (DSC).

For comparison, *tert*-butylcalix[6]arene is less selective breaking the studied guest compounds into two groups: (1) remembered guests inducing formation of metastable polymorphs, and (2) non-remembered guests without such ability (Yakimov et al., 2008). This selectivity of *tert*-butylcalix[6]arene may be used in the analysis of binary mixtures if at least one of their components is from the first group. The efficiency of this analysis was demonstrated using DSC for the binary mixtures with one (Safina et al., 2013) and two (Gabdulkhaev et al., 2016) remembered components.

Guest-induced metastable polymorphs of calixarenes capable of an exothermic solid-phase transition have also a potential in 100% separation of binary mixtures of close homologs (Morohashi et al., 2017; Morohashi and Hattori, 2018) or compounds with close boiling points (Gabdulkhaev et al., 2016).

The phenomenon of polymorphism is more variable than the examples given in this review. In many cases, metastability of a polymorph is in its lower melting point than that of the stable form. Such polymorphs may have more than one melting point with an intermediate exothermic cold crystallization to the more stable forms (Gataullina et al., 2017, 2019). The formation of such polymorphs by guest inclusion and release may be also a kind of molecular recognition when it is selective enough, but in this case the problem is to find sufficient experimental proofs that the host treatment with different guests gives different polymorphs.

## Conclusions

Cooperativity of guest inclusion by solid host with phase transition provides specific types of selectivity for neutral guest compounds that cannot be observed in liquid solutions. In some cases, this selectivity gives practically absolute molecular recognition and may be called smart because it uses the host polymorphism with a very selective and easily detectable memory of the guest included and released. Besides, of the same molecular recognition level is the very selective formation of stable intermediate inclusion compounds, which may be detected by mass-sensitive sensor and in thermogravimetric curves. This process resembles a smart switch of the initial host crystals recognizing only one guest or few guest compounds.

The specific types of structure-property relationships and molecular recognition caused by phase transition at guest inclusion and release may be expected for any solid host capable of clathrate formation. Still, discovery of the host-guest systems with a genuine selectivity for neutral molecules requires an extensive screening, which success cannot be predicted.

## Author Contributions

VG supervised the project and mainly wrote the paper. AG and MZ co-wrote the paper. All authors discussed the reviewed results and commented on the manuscript.

### Conflict of Interest

The authors declare that the research was conducted in the absence of any commercial or financial relationships that could be construed as a potential conflict of interest.
